# Getting Found: Indexing and the Independent Open Access Journal

**DOI:** 10.5811/westjem.2016.6.30836

**Published:** 2016-07-26

**Authors:** Katie Fortney, Linda Suk-Ling Murphy

**Affiliations:** * University of California, California Digital Library, Oakland, California; † University of California, Irvine, Libraries, Irvine, California

Running an independent journal takes much effort, even if only focusing on managing the process of moving articles through the process of submission, review, and publication. Yet publishing an article is not the only goal. Even a great article has little impact unless it can easily be discovered for people to read and cite. Without visibility, even a journal with a terrific editorial board will not get the high quality submissions its editors seek.

The *Western Journal of Emergency Medicine: Integrating Emergency Care with Population Health* (*West*JEM) gets ten times the submissions as a decade ago, and has seen its readership climb. In 2008, *West*JEM averaged 2,907 combined article views and downloads per month. In 2015, the monthly average was 130,000 [Figure]. Without the support of a large publisher, and charging a $400 article processing fee, the journal’s resources are limited. So what has led to the journal’s success? The journal fills a need in an active and growing field, and its editorial board pursues savvy strategies to build strong and sustainable relationships with professional organizations and academic departments.

One crucial piece according to Mark Langdorf, Editor-in-Chief and UC Irvine Professor of Clinical Emergency Medicine, is getting the journal indexed in all major medical databases, and finding sufficient resources.

Bibliographic databases/citation indexes are compilations of descriptive information about books, conference proceedings, reports, journals and the articles they contain. Some are discipline-specific and others cover a broad range of topics. If a journal is well indexed, readers are more likely to discover its content regardless of whether or not the journal itself is known to them.

However, getting a journal indexed can be difficult. While most citation indexes make their journal selection criteria publicly available, those criteria may be hard to meet. Citation indexes compete for users—and usually paid subscribers—the same way journals compete for readers. Most claim that they include more journals than their competitors and offer the *top-tier* journals. Thus, it is in their interest to vet publications carefully. Even if a journal seems eligible for inclusion, it can be labor intensive to bring the journal to the evaluators’ attention and make the case that it should be added to the citation index. *West*JEM applied to be indexed in MEDLINE, the major bibliographic database of the U.S. National Library of Medicine, three times over five years before it was accepted.

Unsurprisingly, working with citation indexes can be a challenge for the journal editorial board. For *West*JEM, its board includes one unusual member: UC Irvine Health Sciences Librarian Linda Murphy. Librarians spend a great deal of time working with citation indexes and often get asked which ones are the best to use and why. They compare the features, scope, and coverage of citation indexes before deciding which to recommend to users engaged in education, research, or patient care. With increasingly tight budgets, librarians have to determine which indexes provide the most cost-effective usage. Because of this multifaceted customer/recommender/user role, librarians are more likely to be able to get the attention of citation indexes and speak their language. Murphy, says Langdorf, has played an essential role in *West*JEM’s indexing success. It is not uncommon for indexing services to ignore messages from the editor and managing associate editor, only to respond when the librarian inquires.

For her part, Murphy says she’s learned much, both from her fellow board members and from the indexing evaluation process. She offers these tips for journal editors who are getting their journal ready for the index submission process:

- Start by identifying relevant databases to be considered for indexing. These are frequently discipline specific.
- Review the citation index’s requirements and its journal evaluation and selection criteria. Each has a publicly available description of their criteria with links to their journal suggestion submission form. Some forms demand more details and are more rigorous, or allow more space to justify inclusion, e.g., MEDLINE Review Application (comprehensive) vs. Thomson Reuters Electronic Journal submission form (limited) vs. Embase Journal Selection Procedures.
- Be persistent, even after a first rejection. Expect the acceptance process to take time. Reconsideration cycles for indexes are measured in years, not months.
- Citation indexes vary in journal coverage and base their selection decisions on a wide variety of criteria. Some focus only on article quality, and require journals to submit issues for them to review. Some want to see international representation on an editorial board, e.g., MEDLINE Journal Selection and Thomson Reuters Journal Selection Process. Scientific journals must also include comprehensive policies on peer-review process, full disclosure of conflicts of interest, and human and animal subjects treatment, among others.
- Establishing an open dialogue with the index editor is crucial. It helps to clarify questions and reasons for rejections and/or reconsideration.
- Sometimes a journal will be required to adjust the way it operates if its goal is to be accepted to a major citation index. For instance, the *West*JEM editorial board was told by both MEDLINE and Web of Science evaluators that the journal had insufficient international focus and submissions by non-U.S. authors. To address this requirement while maintaining writing quality for authors whose first language is not English, *West*JEM has partnered with international emergency medicine organizations to improve the quality of international submissions. MEDLINE also found the journal title too broad, and claimed it already had sufficient journal titles from emergency medicine. So *West*JEM added a subtitle that clarified its niche: “*Integrating Emergency Care with Population Health,*” to set it apart from other specialty journals. Its third MEDLINE application specifically highlighted this population health niche with purposely placed articles demonstrating the journal’s unique role within the larger specialty.
- Journals may get frustrating rejections they might not be able to remedy, e.g., the journal contents are subjects already well represented in the citation index. This is an issue *West*JEM continues to discuss with Thomson Reuters regarding their Science Citation Index. *West*JEM has recently been accepted to a new citation index in the Web of Science Core Collection, *Emerging Sources Citation Index,* and currently is being reconsidered for the Science Citation Index. Inclusion in major indexes is a requirement for some submitting authors, whose university promotions are only supported by publishing in such completely indexed, peer-review journals.
- Being an open access (OA) journal may help. Many citation indexes consider OA journals to be desirable. It is easier to review the journal’s content for quality and has the potential to reach a larger audience worldwide, especially in developing countries that lack resources for expensive subscriptions or per-article purchases. There is also evidence that OA journals garner citations more quickly and broadly than traditional subscription publications, especially in the developing world. Access generates citation.

*West*JEM’s inclusion in MEDLINE (covering issues from 2014 onward) follows its indexing in Scopus (2011), CINAHL (2010), and PubMed (2007), among others. Langdorf and Murphy say the long hours invested have been worth it. What started as a four-page newsletter in 1999, and then became a regional journal, is now a well-established peer-reviewed international journal of some repute. *West*JEM is one of the only three 100% open access MEDLINE indexed emergency medicine journals in the world. The other two are the *Scandinavian Journal of Trauma, Resuscitation and Emergency Medicine* and *BMC Emergency Medicine*. The quantity and quality of submissions is climbing along with their pageviews (on their independent site westjem.com, in eScholarship, and especially on PubMed Central). So are citations to *West*JEM articles. Having achieved inclusion in major citation indexes, the editors are exploring new opportunities such as interactive issues and partnering with Altmetric, a company that provides “*alt*ernative” “social media” *met*rics that scour the Internet to capture article-level activity and immediate impact in blogs, tweets, reader services and news outlets. For an example, see how Altmetric provides data of attention for a recent *West*JEM article, “ The San Bernardino, California, Terror Attack: Two Emergency Departments’ Response,” published in January 2016.

One thing has remained constant during this long decade of growth: The journal remains focused on serving the emergency medicine community. Part of that focus is a commitment to the OA model that ensures everyone working in the field—a specialty that deals with social injustice, health and economic disparities, violence, substance abuse, and disaster preparedness and response—can read the articles. *West*JEM publicly shares and tries to minimize its acceptance-to-publication time so it can spread new knowledge quickly. It maintains a rigorous peer-review process and ethical standards as marked by membership in the Open Access Scholarly Publishers’ Association (OASPA). *West*JEM also waives its article processing fee for authors from low-income countries, and for faculty of departments that support the journal through a membership/print subscription program.

eScholarship, the institutional repository of the University of California system, “*provides a suite of open access, scholarly publishing services and research tools that enable departments, research units, publishing programs, and individual scholars associated with the University of California to have direct control over the creation and dissemination of the full range of their scholarship*.” According to Langdorf, hosting *West*JEM with eScholarship was crucial, especially during early implementation. As the UC Institutional Repository, eScholarship provides valuable support and free services to *West*JEM. These include the peer-review platform, key to *West*JEM’s initial success. Without eScholarship, *West*JEM would never have been started, or continue to grow in the arena of OA publishing.

Many independent journals share similar dedication and willingness to adapt, despite staff turnover, financial constraints, and technology barriers. Nonetheless, the most daunting hurdle can be building the community of authors, reviewers, and readers necessary to establish a strong journal. Perhaps some impressive new technology will come along to solve that problem. In the meantime, indexing—and working with a university librarian—is certainly a wise strategy.

## Figures and Tables

**Figure:**
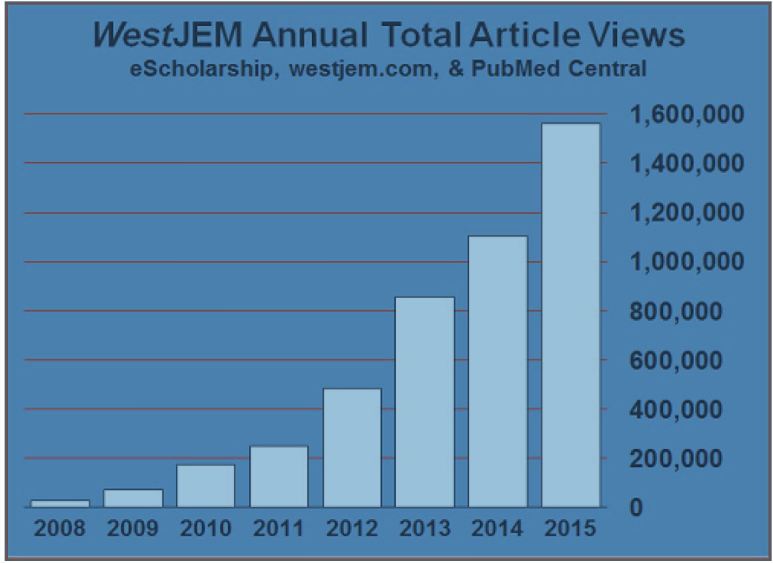
*Western Journal of Emergency Medicine: Integrating Emergency Care with Population Health* (*West*JEM) annual total article page views and downloads since 2008.

